# Prognostic Impact of Spontaneous Conversion to Sinus Rhythm in Patients With Symptomatic Paroxysmal Atrial Fibrillation: A Propensity‐Matched Follow‐Up Study

**DOI:** 10.1111/jce.70128

**Published:** 2025-10-02

**Authors:** Marco Valerio Mariani, Tommaso Recchioni, Nicola Pierucci, Sara Trivigno, Pietro Cipollone, Raffaele Maria Bruti, Domenico Laviola, Marta Palombi, Andrea Matteucci, Agostino Piro, Cristina Chimenti, Gioacchino Galardo, Francesco Pugliese, Carmine Dario Vizza, Carlo Lavalle

**Affiliations:** ^1^ Department of Cardiovascular, Respiratory, Nephrological Aenesthesiological and Geriatric Sciences “Sapienza” University of Rome Rome Italy; ^2^ Clinical and Rehabilitation Cardiology Division, San Filippo Neri Hospital Rome Italy; ^3^ Department of Internal Clinical Aenesthesiological and Cardiovascular Sciences “Sapienza” University of Rome Rome Italy; ^4^ Department of Emergency Critical Care and Trauma Rome Italy; ^5^ Department of General and Specialistic Surgery “Sapienza” University of Rome Rome Italy

**Keywords:** atrial fibrillation, emergency department, spontaneous conversion

## Abstract

**Background:**

Paroxysmal atrial fibrillation (PAF) patients do not invariably progress to persistent AF, with a consistent group of patients showing AF regression during follow‐up. The prognostic implications of spontaneous conversion (SCV) to sinus rhythm (SR) in those patients has never been evaluated yet.

**Objective:**

To evaluate the long‐term prognostic impact of early SCV to SR in patients presenting with symptomatic, hemodynamically stable, PAF in the emergency department (ED).

**Methods:**

Consecutive patients with symptomatic, hemodynamically stable PAF were included. Patients discharged in SR and followed‐up thereafter were stratified based on the occurrence of SCV within 6 h from ED admission. Propensity score matching (PSM) was employed to adjust for relevant baseline differences. The primary outcome was a composite of progression to permanent AF, progression to persistent AF, initiation of antiarrhythmic drugs, AF ablation and need for pharmacologic or electrical cardioversion. Secondary outcomes included AF episodes frequency and individual components of the composite outcome.

**Results:**

Out of 158 patients, 52 experienced SCV while 106 required active cardioversion. After PSM, 52 matched pairs were analyzed. During a median follow‐up of 17.0 months (IQR: 12.8–21.3 months), the primary composite outcome occurred significantly less frequently in the SCV group compared to the non‐SCV group (21 vs. 41 events; log‐rank *p* < 0.001). SCV was associated with a significant reduction of primary outcome occurrence (OR 0.361 [95% CI: 0.176; 0.739], *p*‐value 0.005). The SCV group also exhibited a lower arrhythmic burden, with fewer AF episodes (median 1 [3] vs. 2 [4], *p* < 0.001) and reduced need for cardioversion (*p* = 0.008). The primary and secondary outcomes did not differ among SCV and non‐SCV groups in the unmatched population.

**Conclusions:**

SCV in patients with PAF is associated with lower arrhythmic burden and reduced need for rhythm‐control strategies at follow‐up.

## 1 Introduction

Atrial fibrillation (AF) is the most frequent arrhythmia diagnosed in clinical practice [1]. AF is a dynamic disease with structural and electrical remodeling leading to the progression from initially paroxysmal to persistent/permanent AF [2]. The natural progression from paroxysmal AF (PAF) to persistent/permanent AF is linked to worse cardiovascular (CV) outcomes, with increased rates of heart failure (HF), hospitalizations, stroke, and systemic embolisms [3]. Padfield et al. [4, 5] demonstrated that within 10 years of follow‐up, >50% of PAF patients will transition to persistent AF or be dead, and in an analysis of the RACE V trial De With et al. [5] found that 22% of PAF patients during 1‐year follow‐up showed progression to longer AF episodes. Older age, systemic hypertension, valvular heart disease (VHD), left ventricular hypertrophy (LVH) and left atrial dilation were associated with progression to persistent AF [2]. However, PAF patients do not invariably progress to persistent AF, with a consistent group of patients showing AF regression during follow‐up despite similar number of comorbidities as compared with patients experiencing AF progression [4]. Hence, PAF is not one entity and the identification of patients more prone to develop longer AF episodes, higher AF burden, and/or persistent AF would allow tailored and early rhythm control therapies to improve CV outcomes.

Spontaneous conversion (SCV) of AF to sinus rhythm (SR) has been increasingly studied [6], with CV comorbidities and presence of CV disease negatively associated with SCV [7]. The propensity to SCV may reflect the severity of atrial cardiomyopathy and may be associated with PAF burden and AF progression [8, 9]. However, the prognostic significance of early SCV of PAF in relation to AF progression risk and AF burden has never been explored so far.

In this study we explored the prognostic impact of early SCV to SR in patients presenting to the emergency department (ED) with hemodynamically stable, symptomatic PAF.

## 2 Methods

### 2.1 Study Design, Population and Outcomes

We previously published the incidence and determinants of early SCV to SR in consecutive PAF patients admitted with AF to the ED of Policlinico Umberto I Hospital of Rome from January 2020 to July 2023 [10]. Herein, we present an observational, retrospective follow‐up study including the same consecutive PAF patients discharged in SR from the ED/Hospital and subsequently referred to the arrhythmia out‐patient clinic for long‐term management and follow‐up. Study population was divided in two groups according to the occurrence of early SCV during the index AF episode. Patients were managed following current European Society of Cardiology (ESC) guidelines on AF management [3], with either acute rate or rhythm control therapy applicable at treating physician's discretion. The inclusion criteria were: age over 18 years, presence of hemodynamically stable, symptomatic AF that was not treated with rhythm control strategy before presentation at the ED or within 6 h after ED admission. AF was electrocardiographically diagnosed either at ED admission or if the patient was admitted with an ECG showing AF obtained in an outpatient setting, even if the patient converted on his way to the hospital. Moreover, a minimum follow‐up of 6 months from the index AF episode was required for the inclusion in the study. The following exclusion criteria were applied: hemodynamic instability, AF occurring in the context of critical illness (signs of heart failure [HF] or acute coronary syndrome), persistent or permanent AF, rhythm control strategy applied before the presentation at the ED or within 6 h after ED admission, ED/Hospital discharge in AF without spontaneous or active conversion to SR of the index arrhythmic episode. SCV was defined as conversion to SR without any active cardioversion attempt, either pharmacological cardioversion (PCV) or electrical cardioversion (ECV), before ED admission or during a 6 h observation in the ED. Rate control strategy with beta‐blockers, verapamil and/or cardiac glycosides was attempted at treating physician's discretion and was not considered as active cardioversion attempt.

This study was approved by the Institutional Review Boards on Human Research.

### 2.2 Data Collection and Clinical Outcomes

As described in the previous publication [10], following baseline characteristics were retrieved: age, sex, presence of cardiovascular risk factors, history of coronary artery disease (CAD), chronic obstructive pulmonary disease (COPD), concomitant medications including antiarrhythmic and anticoagulant drugs, ECG features, laboratory investigation results, echocardiographic data. Moreover, we collected SCV occurrence, previous AF episodes and cardioversion attempts, previous SCV, the time of symptom/AF onset and treatments received as drugs for rate/rhythm control and cardioversion attempts. At 7 days after ED/Hospital discharge and every 6 months thereafter, patients received an out‐patient visit, 12‐lead ECG and 24 h Holter monitoring at the arrhythmia clinic. Furthermore, a remote follow‐up phone calls was performed every 3 months and during the first in‐office visit patients were instructed to contact the arrhythmia clinic if arrhythmic symptoms were perceived, leading to the prescription of 12‐lead ECG and/or 24 h Holter monitoring.

The primary outcome of the study was a composite of progression to permanent AF, progression to persistent AF, new antiarrhythmic drugs (AADs) prescription, AF ablation and PCV/ECV during follow‐up. Secondary outcomes included the number of AF episodes at follow‐up and each single component of the primary composite outcome.

### 2.3 Statistical Methods

The Shapiro‐Wilk's test was used for the assessment of normal distribution of variables. Categorical variables were expressed as number and percentages. Continuous variables were expressed as mean and standard deviation or median and interquartile range, as needed. Comparisons among variables were made using the Student's *t*‐test for normally distributed continuous variables, whereas categorical variables were compared using the χ2 test and the Fisher exact test, as needed. The Mann–Whitney *U* test was used to assess the differences between variables with a non‐normal distribution. Patients were divided in two cohorts according to the occurrence of early SCV during the index AF episode. Propensity score matching (PSM) was performed with a 1:1 ratio to reduce the imbalance of covariates among groups. The model included the following covariates: age, gender, left ventricular ejection fraction (LVEF), first AF episode, AAD use, left atrial volume index (LAVI), CAD history. The nearest neighbour matching protocol was performed for PSM and a caliper width of 0.1 was used. Kaplan‐Meier analysis was performed and differences in outcome rate among patients with and without early SCV were evaluated by log‐rank test. Univariate and multivariable Cox regression analysis were performed to obtain odds ratio (OR) of the association between study variables and primary endpoint. All the variables with a *p*‐value < 0.1 at the univariate Cox regression analysis were included in the multivariable model.

For all tests, a *p*‐value less than 0.05 was considered statistically significant. The statistical analysis was performed using SPSS version 27.0 for Mac (IBM Software Inc., Armonk, NY, USA) and R statistical software (R Foundation for Statistical Computing, Vienna, Austria).

## 3 Results

Among 748 patients admitted to the ED of Policlinico Umberto I Hospital of Rome between January 2020 to July 2023 with hemodynamically stable and symptomatic AF included in the previous analysis [10], 158 patients were considered eligible for the study. Study flow‐chart and patients’ selection are presented in Figure S1. Mean age was 66.5 ± 19.2 years and 39.9% were female. Out of 158 patients, 52 experienced SCV during the index AF episode whereas 106 patients did not convert spontaneously and were treated respectively with ECV in 31 patients (29.2%), PCV in 45 patients (42.5%) and a combined approach in the last 30 patients (28.3%). As shown in Table 1, in the unmatched population the non‐SCV group showed a significantly lower rate of previous SCV (*p*‐value 0.008), a higher rate of previous ECV/PCV (<0.001) and had more likely experienced a previous AF episode (*p*‐value 0.05). Furthermore, non‐SCV patients were more frequently on AADs at the time of index AF episode (*p*‐value 0.038). After PSM based on age, gender, LVEF, first AF episode, AAD use, LAVI, CAD history, 52 patient pairs were obtained with good covariate distribution between the study groups (Table 1).

**Table 1:** Baseline clinical characteristics, medication use, laboratory tests, atrial fibrillation (AF) history, and outcomes in patients with or without spontaneous cardioversion (SCV).

	Unmatched populations			Matched populations		
	SCV (*n* = 52)	Non‐SCV (*n* = 106)	*p* value	SCV (*n* = 52)	Non‐SCV (*n* = 52)	*p* value
Clinical characteristics						
Age, years (IQR)	68.5 (19.5)	65.5 (19)	0.119	68.5 (19.5)	68 (16)	0.347
Female, *n* (%)	24 (46.2%)	39 (36.8%)	0.259	24 (46.2%)	21 (40.4%)	0.553
LVEF, % (IQR)	55 (3.2)	55 (7.5)	0.645	55 (3.2)	55 (5)	0.798
Indexed LA Volume, mL/mq	34 (10)	35 (11)	0.261	34 (10)	35 (9)	0.327
BMI, (IQR)	26 (4)	26 (4)	0.816	26 (4)	26 (4)	0.704
Comorbidities						
HTN, *n* (%)	34 (65.4%)	65 (61.3%)	0.620	34 (65.4%)	31 (59.6%)	0.543
DM, *n* (%)	6 (11.5%)	14 (13.2%)	0.767	6 (11.5%)	9 (17.3%)	0.402
Dyslipidemia, *n* (%)	15 (28.8%)	28 (26.4%)	0.747	15 (28.8%)	14 (26.9%)	0.827
Current smoker, *n* (%)	7 (13.5%)	7 (6.6%)	0.231	7 (13.5%)	6 (11.5%)	0.767
Family history of CVD, *n* (%)	11 (21.1%)	28 (26.4%)	0.471	11 (21.1%)	14 (26.9%)	0.491
HF, *n* (%)	14 (26.9%)	30 (28.8%)	0.855	14 (26.9%)	14 (26.9%)	1
IHD, *n* (%)	6 (11.5%)	10 (9.4%)	0.680	6 (11.5%)	3 (5.8%)	0.488
Previous TIA/stroke, *n* (%)	1 (1.9%)	4 (3.8%)	0.532	1 (1.9%)	2 (3.8%)	0.557
COPD, *n* (%)	4 (7.7%)	6 (5.7%)	0.730	4 (7.7%)	3 (5.8%)	1
VHD, *n* (%)	1 (1.9%)	5 (4.7%)	0.387	1 (1.9%)	4 (7.7%)	0.169
AF history						
First AF episode, *n* (%)	25 (48.1%)	34 (32.1%)	0.05	25 (48.1%)	17 (32.7%)	0.110
Previous SCV, *n* (%)	18 (34.6%)	17 (16%)	0.008	18 (34.6%)	10 (19.2%)	0.077
Previous ECV/PCV, *n* (%)	10 (35.7%)	46 (74.2%)	< 0.001	10 (35.7%)	31 (59.6%)	< 0.001
Previous AF ablation, *n* (%)	1 (1.9%)	4 (3.8%)	0.532	1 (1.9%)	1 (1.9%)	1
AF symptoms duration, h (IQR)	4 (5)	10 (29)	0.008	4 (5)	8.5 (40.2)	0.010
CHA2DS2VAS score (IQR)	1.5 (1.75)	2 (2)	0.191	1.5 (1.75)	2 82)	0.522
HAS‐BLED (IQR)	0 (1)	1 (1)	0.117	0 (1)	1 (1)	0.396
Medication						
VKA, *n* (%)	4 (7.7%)	1 (0.9%)	0.041	4 (7.7%)	0 (0%)	0.118
LMWH, *n* (%)	0 (0%)	7 (6.6%)	0.096	0 (0%)	4 (7.7%)	0.118
NOAC, *n* (%)	10 (19.2%)	46 (43.3%)	0.003	10 (19.2%)	25 (48.1%)	0.002
Beta blockers, *n* (%)	23 (44.2%)	51 (48.1%)	0.646	23 (44.2%)	27 (51.9%)	0.432
ACE blocker/AT‐2 blocker, *n* (%)	9 (17.3%)	23 (21.7%)	0.519	9 (17.3%)	10 (19.2%)	0.8
MRA, *n* (%)	3 (5.8%)	4 (3.8%)	0.685	3 (5.8%)	2 (3.8%)	0.647
SGLT2i, *n* (%)	5 (9.6%)	12 (11.3%)	0.745	5 (9.6%)	7 (13.4%)	0.539
Statin, *n* (%)	14 (26.9%)	31 (29.2%)	0.761	14 (26.9%)	15 (28.8%)	0.827
AADs, *n* (%)	9 (17.3%)	35 (33%)	0.038	9 (17.3%)	17 (32.7%)	0.070
Amiodarone, *n* (%)	2 (3.8%)	4 (3.8%)	1	2 (3.8%)	3 (5.8%)	1
Flecainide, *n* (%)	7 (13.5%)	22 (20.8%)	0.266	7 (13.5%)	12 (23.1%)	0.205
Propafenone, *n* (%)	0 (0%)	2 (1.9%)	1	0 (0%)	2 (3.8%)	0.495
Laboratory						
Hemoglobin, g/dl (IQR)	14.2 (3)	14.4 (3.1)	0.661	14.2 (3)	14.2 (2.2)	0.946
eGFR, mL/min (IQR)	65 (54)	68 (42)	0.913	65 (54)	69 (51)	0.792
Potassium, mmol/L (IQR)	3.92 (0.51)	3.96 (0.67)	0.831	3.92 (0.51)	3.9 (0.6)	0.962
Sodium, mmol/L (IQR)	140 (5)	140 (4)	0.912	140 (5)	139.5 (4)	0.538
hs‐Troponin T, mcg/L (IQR)	0.013 (0.01)	0.013 (0.016)	0.842	0.013 (0.01)	0.013 (0.016)	0.831
CRP, mg/dl (IQR)	0.25 (0.47)	0.17 (0.42)	0.137	0.25 (0.47)	0.16 (0.44)	0.090
Outcomes						
AF episodes at follow‐up, *n* (%)	27 (51.9%)	50 (47.2%)	0.574	27 (51.9%)	49 (94.2%)	< 0.001
AF episodes at follow‐up, *n* (IQR)	1 (3)	0 (2)	0.552	1 (3)	2 (4)	< 0.001
Progression to permanent AF, *n* (%)	3 (5.8%)	5 (4.7%)	0.719	3 (5.8%)	5 (9.6%)	0.715
Progression to persistent AF, *n* (%)	12 (23.1%)	25 (23.6%)	0.944	12 (23.1%)	25 (48.1%)	0.008
SCV, *n* (%)	12 (23.1%)	18 (34%)	0.217	12 (23.1%)	17 (32.7%)	0.274
ECV or PCV, *n* (%)	12 (23.1%)	25 (23.6%)	0.944	12 (23.1%)	25 (48.1%)	0.008
AADs prescription, *n* (%)	12 (23.1%)	15 (14.2%)	0.161	12 (23.1%)	15 (28.8%)	0.502
AF ablation, *n* (%)	7 (13.5%)	15 (14.2%)	0.906	7 (13.5%)	15 (28.8%)	0.055
Outcome composito, *n* (%)	21 (40.4%)	41 (38.7%)	0.837	21 (40.4%)	41 (78.8%)	< 0.001

*Note:* Data are presented for unmatched and matched populations comparing patients with SCV and non‐SCV. Continuous variables are expressed as median (interquartile range, IQR) and categorical variables as number (percentage).

Abbreviations: AADs, antiarrhythmic drugs; ACE, angiotensin‐converting enzyme; AF, atrial fibrillation; AT‐2, angiotensin II; BMI, body mass index; COPD, chronic obstructive pulmonary disease; CRP, C‐reactive protein; CVD, cardiovascular disease; DM, diabetes mellitus; ECV, electrical cardioversion; eGFR, estimated glomerular filtration rate; HF, heart failure; hs‐Troponin T, high‐sensitivity troponin T; HTN, hypertension; IHD, ischemic heart disease; LMWH, low‐molecular‐weight heparin; LVEF, left ventricular ejection fraction; MRA, mineralocorticoid receptor antagonist; NOAC, non–vitamin K oral anticoagulant; PCV, pharmacological cardioversion; SCV, spontaneous cardioversion; SGLT2i, sodium‐glucose co‐transporter 2 inhibitor; TIA, transient ischemic attack; VHD, valvular heart disease; VKA, vitamin K antagonist.

### 3.1 Study Outcomes

**Figure 1:**
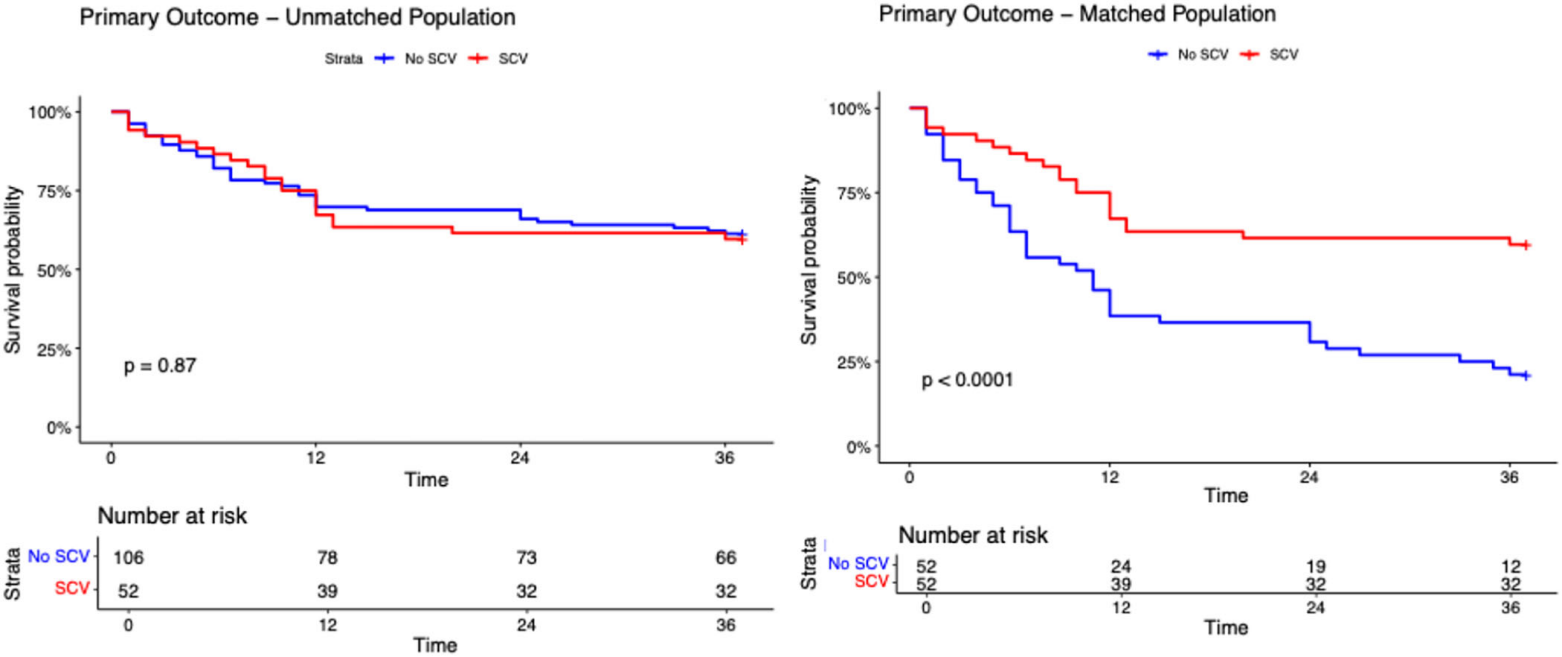
Kaplan–Meier survival curve for the primary composite outcome of progression to permanent AF, progression to persistent AF, new AADs prescription, AF ablation and PCV/ECV during follow‐up in patients with SCV versus no SCV in the unmatched (right panel) and propensity score matched populations. Long‐rank test *p* value and number of patients at risk are shown. SCV, spontaneous cardioversion.

**Figure 2:**
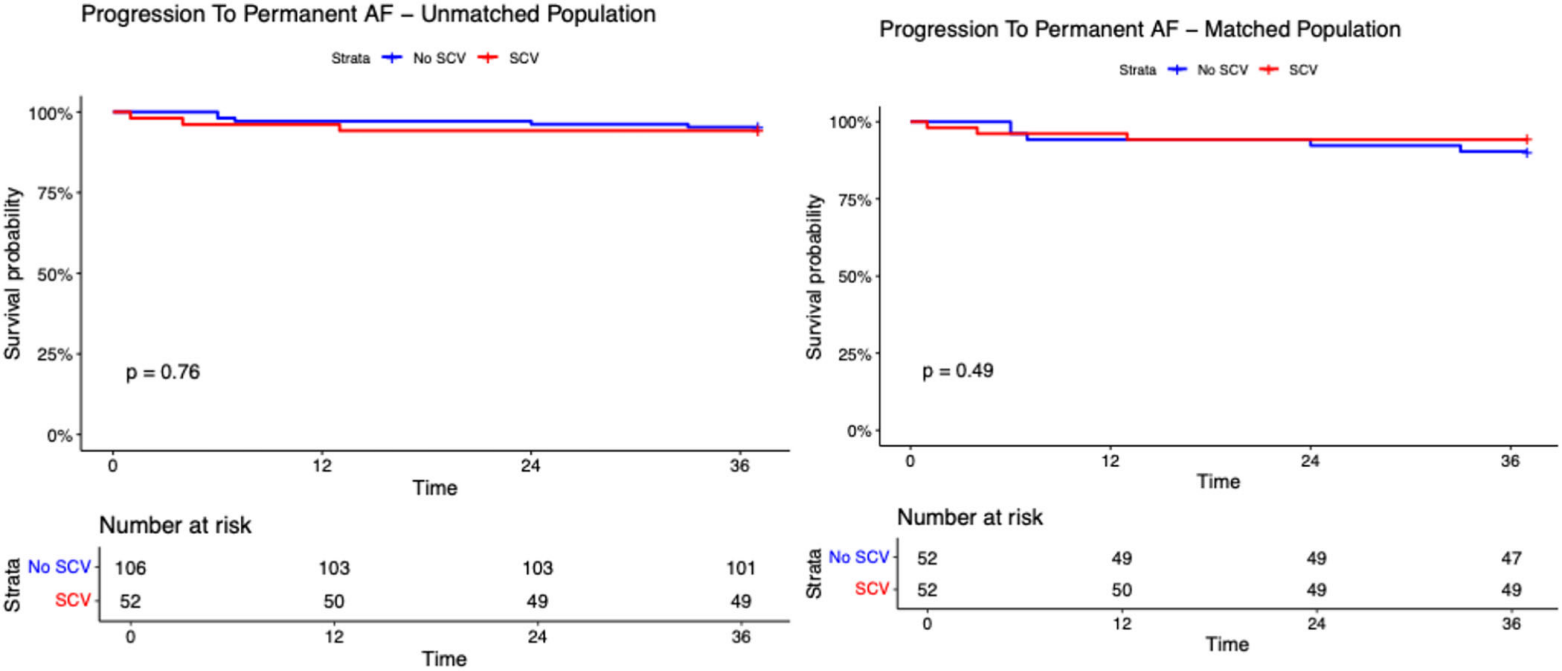
Kaplan–Meier survival curve for the secondary outcome of progression to Permanent AF during follow‐up in patients with SCV versus no SCV in the unmatched (right panel) and propensity score matched populations. Long‐rank test *p* value and number of patients at risk are shown. AF, atrial fibrillation.

**Figure 3:**
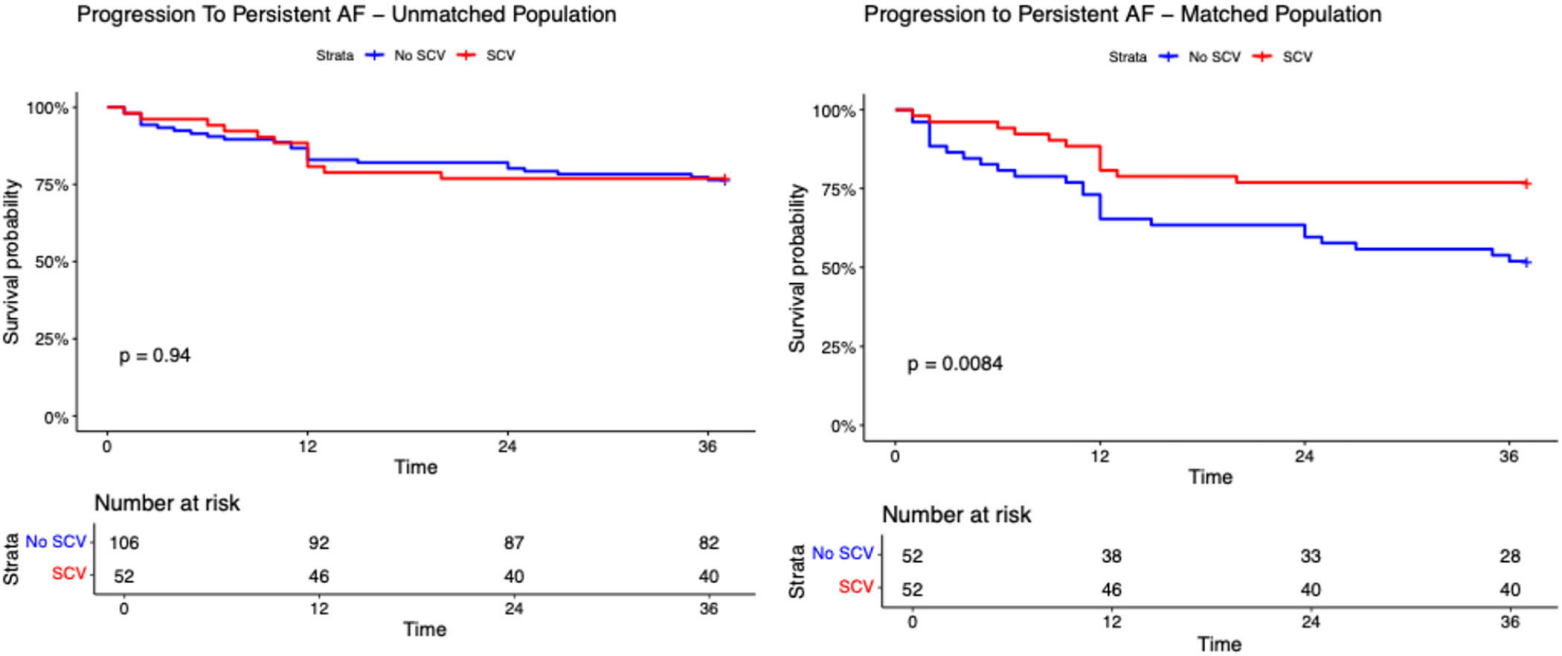
Kaplan–Meier survival curve for the secondary outcome of progression to Persistent AF during follow‐up in patients with SCV versus no SCV in the unmatched (right panel) and propensity score matched populations. Long‐rank test *p*‐value and number of patients at risk are shown. AF, atrial fibrillation.

**Figure 4:**
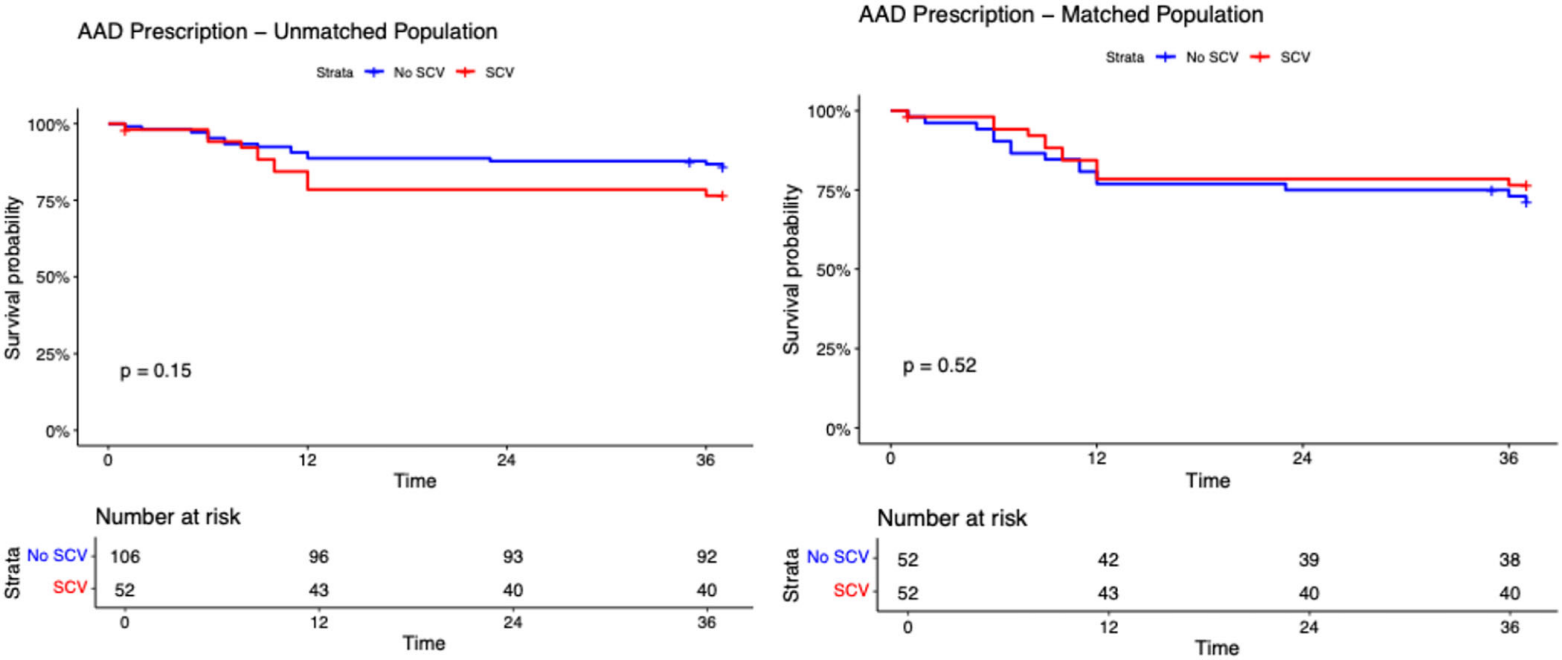
Kaplan–Meier survival curve for the secondary outcome of new AAD prescription during follow‐up in patients with SCV versus no SCV in the unmatched (right panel) and propensity score matched populations. Long‐rank test *p*‐value and number of patients at risk are shown. AAD, antiarrhythmic drug.

**Figure 5:**
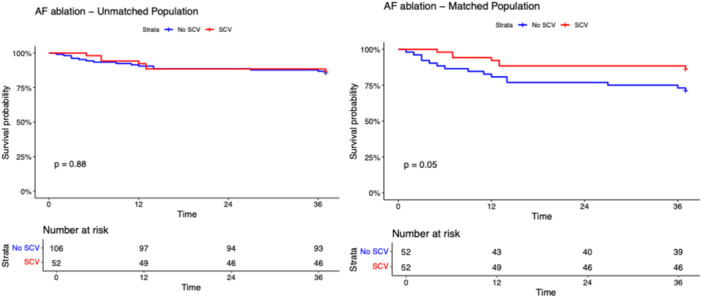
Kaplan–Meier survival curve for the secondary outcome of AF ablation during follow‐up in patients with SCV versus no SCV in the unmatched (right panel) and propensity score matched populations. Long‐rank test *p* value and number of patients at risk are shown. AF, atrial fibrillation.

**Figure 6:**
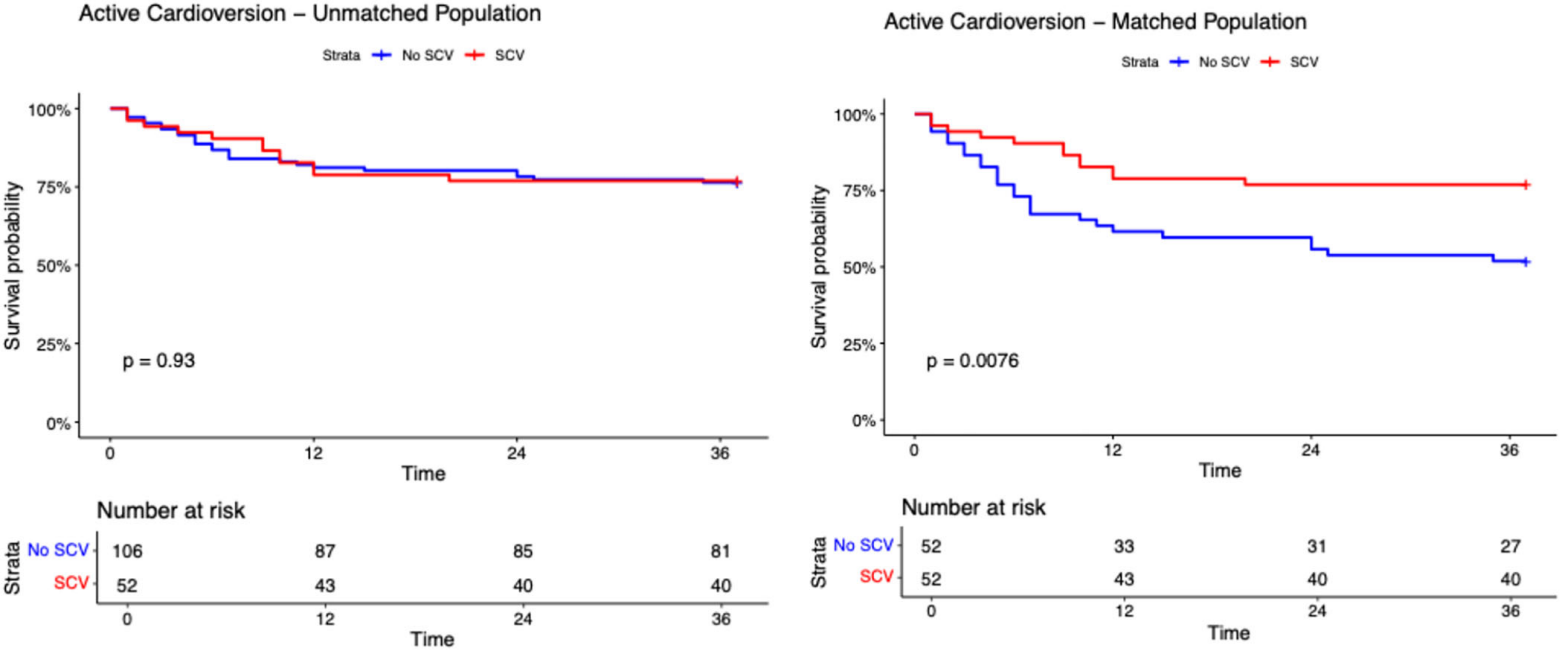
Kaplan–Meier survival curve for the secondary outcome of active cardioversion during follow‐up in patients with SCV versus no SCV in the unmatched (right panel) and propensity score matched populations. Long‐rank test *p* value and number of patients at risk are shown.

After a median follow‐up of 17.0 months (IQR: 12.8–21.3 months), the primary and secondary outcomes did not differ among SCV and non‐SCV groups in the unmatched population (Table 1). The composite outcome occurred in 21 patients in the SCV group and in 41 patients in the non‐SCV group with a long‐rank p‐value 0.870 (Figure 1). As shown in Figures 2, 3, 4, 5, 6 no differences were found in the rates of progression to permanent AF (*p*‐value 0.760), progression to persistent AF (*p*‐value 0.944), new AAD prescription (*p*‐value 0.150), AF ablation (*p*‐value 0.880), and ECV/PCV (*p*‐value 0.930). The absolute and median number of AF episodes were comparable among groups in the unmatched population (*p*‐value 0.574 and *p*‐value 0.552, respectively). In the PSM population, the composite outcome occurred more frequently in the non‐SCV group (*n* = 41) than in the SCV group (*n* = 21), with a statistically significant difference among the groups (log‐rank test *p*‐value < 0.001) (Figure 1). As shown in Table 1, the median number of AF episodes during follow‐up was significantly higher for non‐SCV group as compared with SCV group (2 [5] vs 1 [3] respectively, *p*‐value < 0.001), as well as the absolute number of AF episode (27 in the SCV group vs 49 in the non‐SCV group, *p*‐value < 0.001). No differences among groups were found in the rates of progression to permanent AF (3 in the SCV group vs 5 in the non‐SCV group, log‐rank *p*‐value 0.484; Figure 2) and new AAD prescription (12 in the SCV group vs 15 in the non‐SCV group, log‐rank *p*‐value 0.525; Figure 4). A nonsignificant trend towards higher rate of AF ablation at follow‐up was detected in the non‐SCV group (7 in the SCV group vs 15 in the non‐SCV group, log‐rank p‐value 0.050; Figure 5). Significantly higher rates of progression to persistent AF and ECV/PCV were detected in the non‐SCV group during follow‐up (log‐rank *p*‐value 0.008 and *p*‐value 0.008 respectively, Figures 3 and 6). As shown in Table 2, at the Cox regression analysis SCV occurrence resulted the only factor independently associated with the primary outcome both in the univariate (OR: 0.358 [0.211; 0.608], *p*‐value < 0.001) and in the multivariable analysis (OR: 0.361 [0.176; 0.739], *p*‐value 0.005). In the univariate Cox regression analysis, none of the independent variables resulted significantly associated with the primary outcome in the unmatched population (Table 3).

**Table 2:** Univariate and multivariable Cox regression analysis for the primary study outcome in the matched population.

Univariate cox regression analysis			Multivariable cox regression analysis	
Variable	OR (95% CI)	*p* value	OR (95% CI)	*p* value
Age	0.998 (0.979; 1.016)	0.814		
Female sex	0.794 (0.478; 1.320)	0.374		
HTN	0.824 (0.497; 1.365)	0.452		
DM	1.032 (0.509; 2.092)	0.931		
IHD	0.515 (0.245; 1.084)	0.081	0.614 (0.231; 1.636)	0.330
LVEF	0.933 (0.864; 1.007)	0.074	0.936 (0.860; 1.017)	0.118
eGFR	1.002 (0.987; 1.017)	0.780		
**SCV**	**0.358 (0.211; 0.608)**	**< 0.001**	**0.361 (0.176; 0.739)**	**0.005**
First AF episode	0.868 (0.520; 1.447)	0.586		
LAVI	0.988 (0.962; 1.014)	0.369		
BMI	1.031 (0.937; 1.135)	0.525		

Abbreviations: AF, atrial fibrillation; BMI, body mass index; CI, confidence interval; DM, diabetes mellitus; eGFR, estimated glomerular filtration rate; HTN, hypertension; IHD, ischemic heart disease; LAVI, left atrium volume indexed; LVEF, left ventricular ejection fraction; OR, odd ratio; SCV, spontaneous cardioversion.

**Table 3:** Univariate cox regression analysis for the primary study outcome in the unmatched population.

Univariate cox regression analysis		
Variable	OR (95% CI)	*p* value
Age	1.007 (0.989; 1.026)	0.461
Female sex	0.997 (0.600; 1.656)	0.990
SAH	0.823 (0.497; 1.364)	0.451
DM	1.243 (0.613; 2.520)	0.546
CAD history	1.384 (0.659; 2.909)	0.391
LVEF	0.987/0.931; 1.047)	0.666
eGFR	1 (0.982; 1.018)	0.987
SCV	0.962 (0.568; 1.628)	0.885
First AF episode	0.782 (0.469; 1.304)	0.346
Indexed LA Volume	0.986 (0.960; 1.013)	0.319
BMI	0.905 (0.810; 1.012)	0.081

Abbreviations: AF, atrial fibrillation; BMI, body mass index; CI, confidence interval; DM, diabetes mellitus; eGFR, estimated glomerular filtration rate; HTN, hypertension; IHD, ischemic heart disease; LAVI, left atrium volume indexed; LVEF, left ventricular ejection fraction; OR, odd ratio; SCV, spontaneous cardioversion.

## 4 Discussion

Atrial fibrillation is a widespread arrhythmia, with a dynamic progression over time from paroxysmal AF to persistent/permanent AF due to structural and electrical remodeling. Such progression finally leads to worse CV outcomes, increased rates of HF, hospitalizations, stroke and systemic embolisms [1, 3]. In this observational, retrospective and propensity‐matched study of patients presenting to the ED with symptomatic and hemodynamically stable PAF, we aimed to investigate the long‐term prognostic impact of early SCV to SR. Our main findings pointed out how SCV is associated with a significantly lower rate of the composite outcome of progression to permanent AF, progression to persistent AF, new AADs prescription, AF ablation and PCV/ECV during an at least 6 months follow‐up in the PSM population (21 vs 41 patients, log‐rank test *p*‐value < 0.001). Although the primary and secondary outcomes did not differ among SCV and non‐SCV groups in the unmatched population, this study confirms the heterogeneity of PAF as a clinical entity. Despite similar baseline characteristics, in fact, the PSM patients who experienced SCV had significantly fewer AF episodes during follow‐up (median: 1 [3] vs. 2 [5]; *p* < 0.001) and a lower requirement for cardioversion (*p* = 0.008), suggesting a less aggressive disease trajectory. These findings are consistent with the growing body of literature highlighting that AF progression is not uniform [4] and that early AF patterns can offer prognostic value for subsequent clinical evolution [4, 11].

Recent data from the RACE V study and from the large Blum's et al. systematic review and meta‐analysis, supports the hypothesis that paroxysmal AF includes several phenotypes and patients with longer episodes and higher AF burden tends to have more severe underlying comorbidities [5, 12]. Our data emphasise these observations, suggesting that patients with SCVs may suffer of a milder AF phenotype, potentially reflecting less atrial electro‐mechanical remodelling and therefore a lower burden of atrial cardiomyopathy. Furthermore, the clinical features of patients’ cohort experiencing SCV reflects the available registries trends, such as the Canadian AF Registry, showing that progression to persistent AF is strongly associated with underlying comorbidities such as mitral regurgitation, left atrial enlargement and aging [4].

Although our study did not find significant differences between the cohorts (SCV and n‐SCV) in terms of progression to permanent atrial fibrillation or the onset of AAD—both in the PSM and unmached population—a non‐statistically‐significant trend in favor of the n‐SCV cohort was observed. Progression to permanent atrial fibrillation occurred specifically in about 5% of the patients, showing a lower incidence if compared to data published by longer‐term and larger registries such as CARAF, reporting AF progression rates of 8.6 per 100 patient‐years [4]. Of course this mismatch may be a consequence of several factors: on the one hand, our median follow‐up duration was quite shorter (17 months), thus probably limiting our opportunity to detect the stepwise disease transition. On the other hand, it highlights a crucial fact: our population had likely less advanced underlying heart disease, as reflected by a simple but relevant comparison; 69% of CARAF patients had, in fact, a LVEF < 40%, whereas the median LVEF of our patients was 55%.

With our study, we join the growing scientific debate on the role of arrhythmic substrate within rhythm control. Emerging evidence from randomized controlled trials and observational cohorts supports early interventions to prevent progression and improve outcomes [13]. In this context, an early SCV may represent a surrogate marker of favorable atrial substrate, potentially identifying a subgroup of patients with a better arrhythmic prognosis and, indeed, “hard endpoints.” Conversely, the absence of SCV may help identifying patients who could benefit from early and aggressive rhythm control strategies, including substrate mapping‐guided ablation and aggressive interventions on cardiovascular risk factors. Not only the SCV “*per se”* but also the temporal pattern of AF is, indeed, a true reflection of patient's comorbidities [5]; nevertheless, patients with the same comorbidities—and thus potentially the same substrate‐ may exhibit different PAF trajectories, thus suggesting how there may be a complex and still unclear interplay between triggers and substrate. Certainly, patients experiencing longer AF episodes might show a more advanced substrate, whereas patients with shorter episodes and SCV, in whom triggers are dominant, might have a healthy substrate and better prognosis.

As already shown in the REVERSE‐AF study, patients trending to permanent AF are often comorbid, older and frail, and modifying interventions on cardiovascular risk factors are effective in reducing AF progression [14]. Therefore, SCV appears to be more than a benign and spontaneous phenomenon. Instead, it may reflect an inherently different structural phenotype of AF, with significant prognostic implications. SCV could serve as an early and noninvasive predictor of a favorable disease trajectory and help in tailoring AF management in the acute and chronic setting. As a result, a “wait‐and see” approach for SCV of symptomatic, hemodynamically stable PAF in the ED may not only avoid unnecessary early active cardioversion but may also provide further prognostic information useful for the definition of a personalized treatment strategy [15]. Future prospective, randomized, and controlled studies are needed to validate SCV as a clinical tool in risk stratification and to explore its integration within early rhythm management algorithms.

### 4.1 Limitations

This study has several limitations. First, its retrospective design and single‐center setting may limit generalizability. Second, although propensity score matching was used to minimize baseline imbalances, residual confoundings cannot be excluded. Third, SCV was defined within a 6 h ED window, which may have excluded late converters with similar phenotypes. Fourth, the follow up design of the study, based on phone monitoring or face‐to‐face visits only, may have significantly reduced the detection of asymptomatic AF episodes and AF progression, despite the underreporting phenomenon should affect similarly both the study groups. Fifth, the small sample size may limit the study results that need to be confirmed in larger studies. Lastly, the relatively short median follow‐up period may have limited the detection of late‐onset AF progression or clinical events.

## 5 Conclusions

SCV in patients with PAF is associated with lower arrhythmic burden and reduced need for rhythm‐control strategies at follow‐up. The possible role of SCV as a noninvasive prognostic indicator useful to stratify patients and guide tailored therapeutic strategies needs further investigation in larger and randomized trials.

## Conflicts of Interest

The authors declare no conflicts of interest.

## Supporting information


**Figure S1**: Study flow‐chart and patients' selection.

## Data Availability

The data that support the findings of this study are available on request from the corresponding author. The data are not publicly available due to privacy or ethical restrictions.
