# Supplementary material for: Prognostic Impact of Spontaneous Conversion to Sinus Rhythm in Patients With Symptomatic Paroxysmal Atrial Fibrillation: A Propensity‐Matched Follow‐Up Study
Source: J Cardiovasc Electrophysiol. 2025 Oct 2;36(12):3222–30. doi: 10.1111/jce.70128 (PMC12697228; doi:10.1111/jce.70128)
Supplement: Supplementary file 1 — Figure S1: Study flow‐chart and patients' selection. [file JCE-36-3222-s001.docx]

**
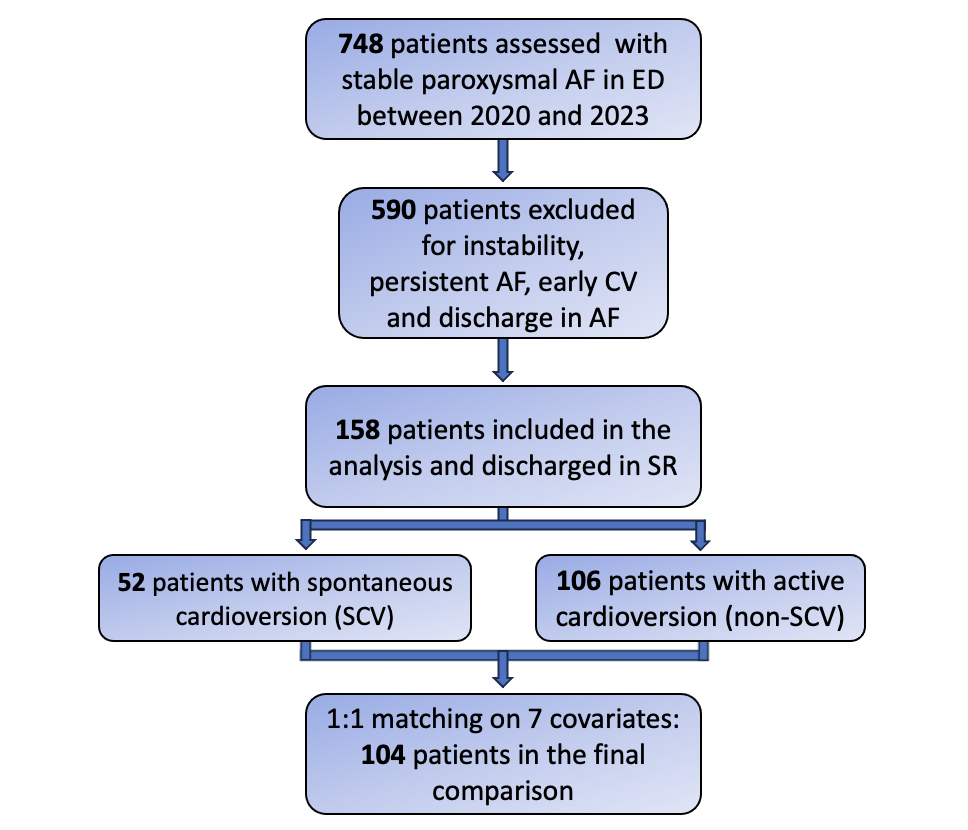
**

**Figure S1. Study flow-chart and patients’ selection.** AF: atrial fibrillation; CV: conversion; ED: emergency department; SCV: spontaneous conversion; SR: sinus rhythm.
